# Characterization of a bacterial strain T226 and its efficacy in controlling post-harvest citrus anthracnose

**DOI:** 10.3389/fmicb.2025.1678436

**Published:** 2025-10-13

**Authors:** Qian Wang, Juan Song, Quan Zeng, Xuelian Ye, Guoying Shi, Chunjin Hu

**Affiliations:** Microbiology Research Institute, Guangxi Academy of Agricultural Sciences, Nanning, Guangxi, China

**Keywords:** citrus anthracnose, antagonistic bacteria, 16S rDNA, *Lysobacter enzymogenes*, control effect

## Abstract

Postharvest anthracnose of citrus, caused by the fungus *Colletotrichum gloeosporioides*, leads to significant economic losses. There is a growing need for effective biological control agents to manage this disease. In this study, a bacterial strain (T226) with antagonistic potential was isolated from sugarcane rhizosphere soil. It was identified as *Lysobacter enzymogenes* through a polyphasic approach, encompassing 16S rDNA phylogenetic analysis (with 100% bootstrap support), morphological observation, and physiological and biochemical characterization. Strain T226 demonstrated strong and stable antagonistic activity against *C. gloeosporioides*. Its efficacy remained consistent over eight continuous subcultures on artificial media, indicating high metabolic stability. *In vivo* biocontrol assays confirmed its potency. Specifically, artificial stab inoculation tests showed a disease control efficacy of 78.2%, while under conditions of natural infection, T226 achieved control efficacies of 61.3% at 20 and 60 days post-application. To our knowledge, this is the first report identifying *Lysobacter enzymogenes* as a highly effective biocontrol agent against citrus postharvest anthracnose. The high stability and significant efficacy of strain T226 underscore its considerable potential for practical application in postharvest protection during citrus storage.

## 1 Introduction

Citrus (*Citrus reticulata* Blanco) is one of the world’s most economically important fruit crops, with global production exceeding one million tons annually (FAO data). However, significant postharvest losses occur because of physiological deterioration and pathogenic infections during storage and transport (Spadaro et al., 2021). Among the major postharvest diseases, anthracnose, primarily caused by the fungus *Colletotrichum gloeosporioides* (Penz.) Sacc., poses a serious threat to citrus fruit quality and marketability worldwide (Huang et al., 2013). Notably, *C. gloeosporioides* can infect fruit both pre- and post-harvest. Pre-harvest infections often occur through wounds (e.g., from hail, insects, or mechanical injury) and remain latent, becoming symptomatic only after harvest under favorable conditions of high humidity and temperature during storage or ripening (Zhang et al., 2018; Peres et al., 2005). This latent nature complicates disease management and contributes to substantial economic losses (Zhou et al., 2025; Acedo, 2019).

While synthetic fungicides remain a primary control strategy for postharvest diseases, concerns over fungicide residues on fruit, the development of pathogen resistance, and increasing consumer demand for residue-free produce have driven the search for safer, eco-friendly alternatives (Droby et al., 2016; Francesco et al., 2016). Biological control using antagonistic microorganisms represents a promising approach for managing postharvest diseases (Zhu et al., 2025; Tian et al., 2020).

Significant progress has been made in identifying biocontrol agents against common citrus postharvest pathogens like *Penicillium digitatum*, *Penicillium italicum* (causing green and blue mold), *Alternaria alternata* (causing Alternaria rot), and *Geotrichum candidum* (causing sour rot) (Liu et al., 2023a, 2023b; Zhu et al., 2019). However, research focused specifically on biocontrol of postharvest citrus anthracnose caused by *C. gloeosporioides* is comparatively limited (Silue and Fawole, 2024; Papoutsis et al., 2019). Although some studies have reported potential antagonists like certain *Brevibacillus* and *Lactobacillus* strains (Liu et al., 2023a, 2023b), studies that comprehensively identify and evaluate effective, stable biocontrol agents for this disease, particularly from under-explored bacterial genera, are needed. Anthracnose caused by *C. gloeosporioides* is also a major constraint for other subtropical fruits, highlighting the broader relevance of finding effective biocontrol solutions.

Bacteria of the genus *Lysobacter*, commonly found in soil and water environments, are increasingly recognized for their potent antagonistic activity against diverse plant pathogens, including fungi, oomycetes, bacteria, and nematodes (Christensen and Cook, 1978). Their biocontrol potential is attributed to the production of lytic enzymes (chitinases, glucanases, proteases), antibiotics (e.g., HSAF, WAP-8294A2), and other bioactive compounds, coupled with competitive colonization abilities (Francesca et al., 2021). Several *Lysobacter* species and strains (e.g., *L. enzymogenes* strains C3 and OH11) have demonstrated efficacy against important plant diseases such as turfgrass brown patch, wheat scab, tomato bacterial wilt, and pepper *Phytophthora* blight, achieving control efficacies often exceeding 80% (Yang et al., 2023; Chen et al., 2018; Qian et al., 2009). These characteristics make *Lysobacter* spp. promising candidates for biological control. Despite this potential, no strains of *Lysobacter*, and specifically *L. enzymogenes*, have been reported for the control of citrus anthracnose, either pre- or post-harvest.

During a screening for potential biocontrol agents against citrus postharvest diseases, bacterial strain T226 was isolated from the rhizosphere soil of sugarcane in Nanning, Guangxi, China. Preliminary tests indicated strong *in vitro* antagonism against *C. gloeosporioides*. Therefore, the specific objectives of this study were to: (i) comprehensively identify strain T226 using a polyphasic approach combining 16S rDNA gene sequencing with morphological, physiological, and biochemical characterization; (ii) evaluate the *in vitro* antagonistic stability of strain T226 against *C. gloeosporioides* over multiple subcultures; (iii) assess the *in vivo* efficacy of strain T226 in controlling citrus postharvest anthracnose under both artificial inoculation and natural infection conditions.

## 2 Materials and methods

### 2.1 Test strains and culture medium

Bacterial biocontrol T226 was isolated from sugarcane (*Saccharum officinarum* L.) rhizosphere soil in Nanning city, Guangxi, China (23°14′ N, 108°03′ E). Soil samples were serially diluted (10^−2^ to 10^−5^) in sterile water, plated on Nutrient Agar (NA; HopeBio, Qingdao, China), and incubated at 30 °C for 48 h. Colonies exhibiting antifungal activity against *C. gloeosporioides* were purified by streak-plating three times. One dominant antagonistic strain, designated T226, was selected for further study.

A total of 14 plant pathogens were used in this study to evaluate the antagonistic spectrum of strain T226. They included 10 fungi: *C. gloeosporioides* “A”; *C. gloeosporioides* “B”; *C. musae*; *Rhizoctonia solani*; *Exserohilum turcicum*; *Cochliobolus heterostrophus*; *Sclerotium rolfsii*; *Alternaria alternata*; *Curvularia lunata*; *Alternaria musae*; and four bacterium: *Xanthomonas oryzae* pv. *oryzae*; *Xanthomonas campestris* pv. *citri*; *Ralstonia solanacearum*; *Pectobacterium carotovorum* pv. *carotovorum* dye; all pathogenic strains were obtained from and preserved by the Culture Collection of the Institute of Microbiology, Guangxi Academy of Agricultural Sciences, Nanning, China. Fungi were cultured on Potato Dextrose Agar (PDA; BD Difco at 28 °C for 5–7 days), bacteria were cultured in Nutrient Agar (NA; HopeBio, Qingdao, China) at 30 °C for 48 h.

### 2.2 Antibacterial activity of strain T226

#### 2.2.1 Antagonistic effects and inhibition spectrum of strain T226 against citrus postharvest anthracnose

To test the inhibitory activities of antagonistic bacteria against citrus anthracnose, plate confrontation assays were performed. Mycelial plugs (diameter 5 mm) in the centers of Petri dishes were inoculated, then cultured for 7 days. About 3 cm from the mycelial plug, the antagonistic strain T226 cultured for 48 h was streaked with the inoculation ring, and a thin line of 3 cm was drawn on the plate symmetrically. Control plates were inoculated only with the pathogen mycelial plug. All plates were cultured at 28 °C. Plates were replicated three times.

When control plates were covered with a dish, citrus anthracnose colony diameter was measured. Colony growth inhibition rate was calculated as follows:


$$\begin{array}{l} \text{Colony growth inhibition rate}\;(\%)=\\ \frac{\text{Control colony}\;\mathrm{net}\;\text{growth distance}-\mathrm{Net}\;\text{growth distance of treated colonies}\;}{\text{Control colony}\;\mathrm{net}\;\text{growth distance}}\times 100 \end{array}$$

The antibacterial spectrum of strain T226 against 10 plant pathogenic fungi and four plant pathogenic bacteria, plate confrontation assays were performed.

#### 2.2.2 Detection of antibacterial activity of fermentation broth of strain T226

Strain T226 was activated in nutrient broth liquid medium. Activated bacterial suspension was added to new nutrient broth medium at a volume ratio of 1:60, and cultured in a shaker (190 r/min) at 30 °C for 2 days. These parameters were selected based on a protocol optimized for antimicrobial compound production in closely related *Lysobacter* spp. strains (Yang et al., 2023), and were validated for T226 in our preliminary experiments, which resulted in high cell density and consistent antibacterial activity. Three fermentation broths were prepared: A (fermentation broth), B (high-temperature sterilized fermentation broth), C (centrifuged fermentation broth to obtain the supernatant which was passed through a bacterial filter) (Wang et al., 2010). Samples (15 μL) of each fermentation broth were added to sterile filter paper (6 mm diameter); sterile water was used as blank control. The antibacterial activity of different fermentation broths was determined by plate confrontation assay using *C. gloeosporioides* as an indicator bacterium.

#### 2.2.3 Effect of antagonistic bacteria on mycelial growth of pathogens

The morphological differences between the mycelia at the edge of the inhibited colony and the normal mycelia at the edge of the control colony were observed under an optical microscope using the confrontation culture method.

### 2.3 Assessment of the stability of antibacterial activity after subculturing

The phenotypic stability of the antibacterial activity of strain T226 was assessed through successive subculturing. The strain was continuously transferred and cultured on NA slant medium eight times, with each transfer occurring every 15 days. After each transfer, it was cultured at 30 °C for 48 h and then stored at 4 °C. After the eighth transfer, the antibacterial activity against the target pathogen was measured using the plate confrontation assay to determine if the inhibitory capability was maintained. The stability test was performed three independent biological replicates.

### 2.4 Control effect of strain T226 on postharvest citrus anthracnose

#### 2.4.1 Determining the control effect of fruit inoculated with pathogens

Fresh citrus fruits were collected. A wound (3 × 3 mm) was made at the waist of the fruit, and 20 μL of biocontrol bacteria suspension (2 × 10^8^ CFU/mL) was inoculated in the wound. Sterile water was used as a blank control; 200 mg/L prochloraz was used as a positive control. After inoculation, fruits were placed into a plastic box and stored at 25 °C. After 24 h, 20 μL of 10^4^/mL conidia suspension of *C. gloeosporioides* was inoculated into the wound. The plastic box was placed within a plastic bag, and relative humidity was maintained at approximately 95%. After storage at 28 °C for 24 h, observation began; fruit were observed daily. Fruit rot rate and a disease index of citrus anthracnose were monitored. Each treatment contained 15 fruits, and was replicated three times.

Citrus fruit disease grades were 0, 1, 3, 5, 7, and 9, where lesions covered <0, 5, 6–10%, 11–25%, 26–50%, and >51% of the fruit area, respectively (Droby et al., 2002).


$$\text{Rotting}-\mathrm{pod}\;\text{rate}\;(\%)=\;\frac{\text{Number of rotten fruit}}{\text{Total of fruit}}\times 100$$

$$\begin{array}{l} \text{Disease index}=\\ \frac{\sum (\text{Number of rotten fruits}\;\mathrm{at}\;\mathrm{all}\;\text{level}\;\mathrm{sit}\times \text{The representative value of this level})}{\text{Total of fruit}\times \text{The highest representative value}}\times 100 \end{array}$$

$$\begin{array}{l} \text{Efficacy}\;(\%)=\\ \frac{\text{Comparison of disease index}-\text{Treatment disease index}}{\text{Comparison of disease index}}\times \;100 \end{array}$$

#### 2.4.2 Control test of antagonistic bacteria treatment on natural citrus fruit disease

Citrus fruits were soaked in three treatments (2 × 10^8^ cfu/mL antagonistic bacteria culture diluent, 200 mg/L prochloraz, sterile water control) for 2 min, dried completely and packaged with polyethylene film. Fruits were then stored in cartons at room temperature. After disease spots first appeared, the incidence and disease index of fruit were measured daily. Each treatment contained 15 fruit and was replicated three times. Disease classification standards and formulae used in calculation are as detailed in section 2.4.1.

### 2.5 Identification of strain T226

The identification of strain T226 was performed using a polyphasic approach. First, morphological characteristics (including cell shape, size, flagellation, and colony morphology on NA medium), physiological traits (e.g., growth temperature range, pH tolerance, NaCl tolerance), and biochemical properties (e.g., Gram staining, oxidase, catalase, hydrolysis of starch, utilization of various carbon sources and nitrogen sources) were examined according to standard methods as described in “Common bacterial system identification manual” (Dong and Cai, 2001) and “Bergey’s Manual of Systematic Bacteriology” (Buchanan, 1984). DNA was extracted by lysis, using primers PF1 (5′-AGAGTTTGATCATGGCTCAG-3′) and PR1 (5′-TACGGTTACCTTGTTACGACTT-3′) for PCR amplification. The PCR reaction system (75 μL) included: dNTP Mixture (37.5 μL), PF1 (2.5 μL), PR1 (2.5 μL), template DNA (2.5 μL), and ddH_2_O (30 μL). PCR reaction conditions involved: 94 °C for 3 min, 30 amplification cycles (94 °C for 55 s, 50 °C for 50 s, and 72 °C for 1 min 10 s), and a final extension at 72 °C for 10 min. After the reaction, 5 μL of solution was mixed with 1 μL of 6 × loading buffer and detected by 1% agarose gel electrophoresis (Wang et al., 2021). Purified PCR amplification products were sent to Shanghai Shenggong Bioengineering Technology Service Co., Ltd. for sequencing. Sequencing results were submitted to a BLAST search.^1^ MEGA version 11.0 (Tamura et al., 2021) was used to construct a phylogenetic tree based on the 16S rDNA sequence of the T226 strain and those of related bacteria from GenBank.

### 2.6 Data statistics and analysis

The significant differences among treatments were analyzed using Duncan’s new multiple range test in the software DPS 9.1 (v9.1). Differences were considered statistically significant at *p* < 0.05.

## 3 Results

### 3.1 Antibacterial activity of strain T226

#### 3.1.1 Antagonistic effect of strain T226 against *Colletotrichum gloeosporioides* and its antifungal spectrum

**Figure 1:**
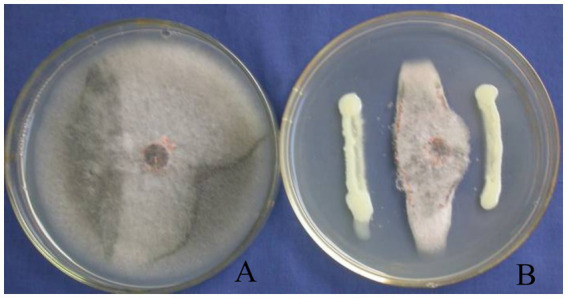
Growth inhibition of *C. gloeosporioides* by biocontrol strain T226. **(A)** Control. **(B)** Strain T226.

**Figure 2:**
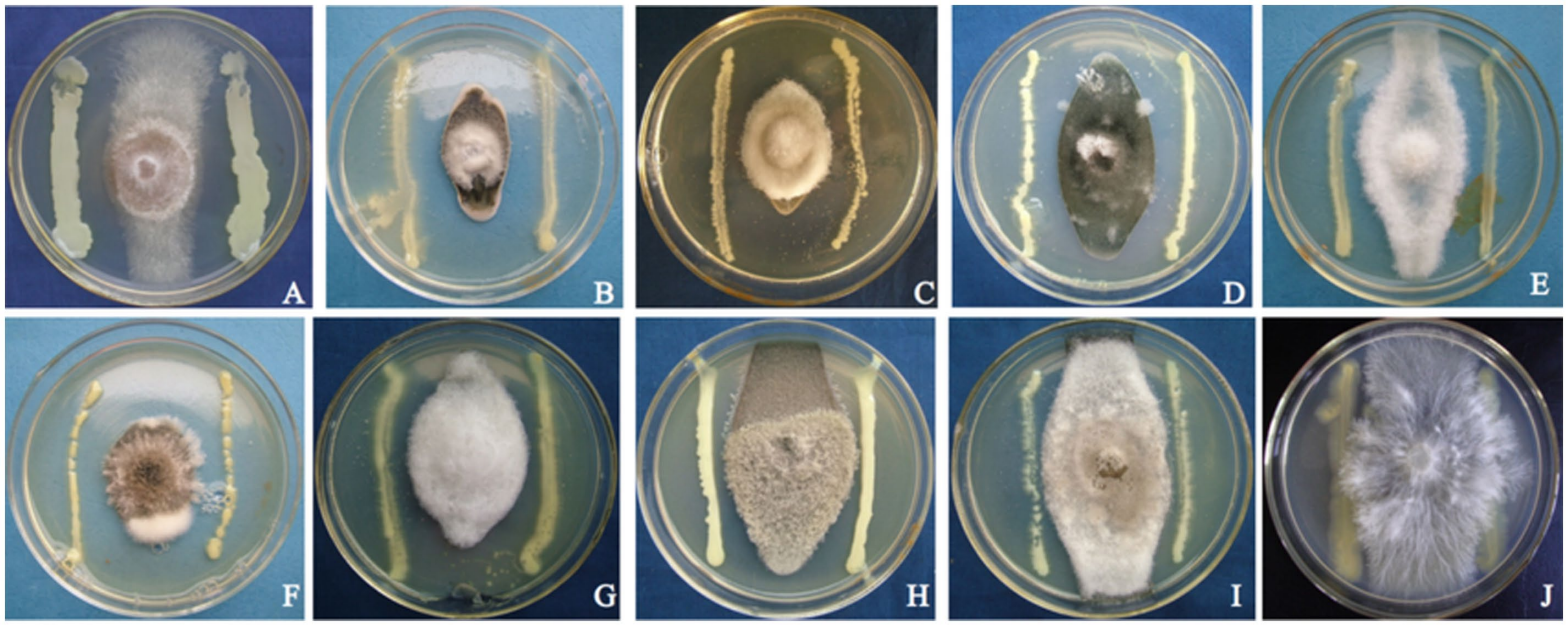
Antagonistic photos of strain T226 against some pathogenic fungi. **(A)** *Rhizoctonia solani*. **(B)** *Colletotrichum gloeosporioides* “B”. **(C)** *Colletotrichum gloeosporioides* “A”. **(D)** *Cochliobolus heterostrophus*. **(E)** *Colletotrichum musae*. **(F)** *Exserohilum turcicum*. **(G)** *Alternaria alternata*. **(H)** *Alternaria musae*. **(I)** *Curvularia lunata*. **(J)** *Sclerotium rolfsii*.

**Figure 3:**
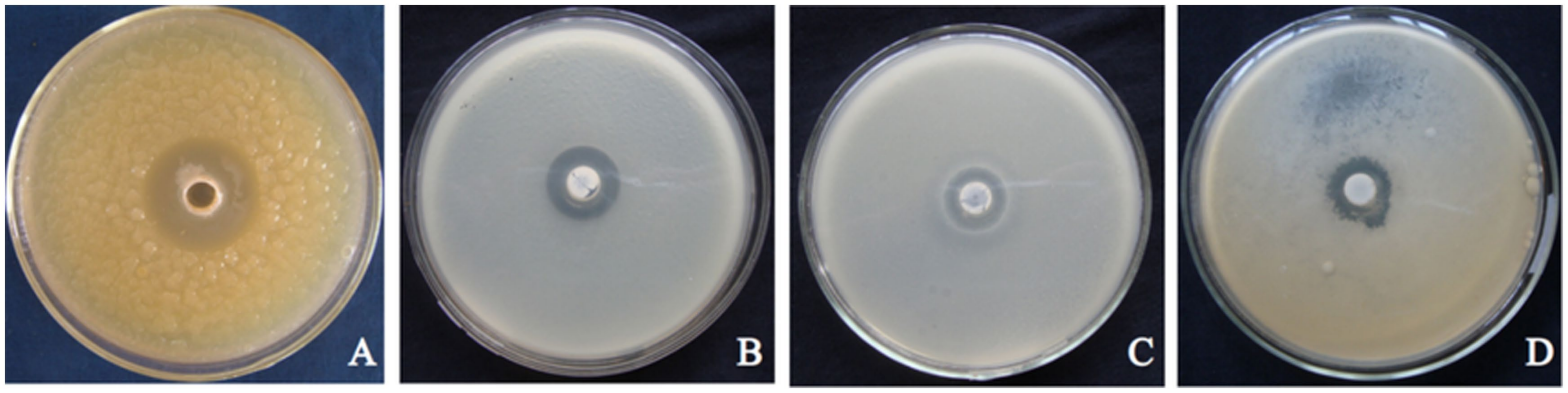
Antagonistic photos of strain T226 against some pathogenic bacteria. **(A)** *Xanthomonas campestris* pv. *citri*. **(B)** *Xanthomonas oryzae* pv. *oryzae*. **(C)** *Ralstonia solanacearum*. **(D)** *Pectobacterium carotovorum* pv. *carotovorum*.

Strain T226 had significant antibacterial activity against *C. gloeosporioides*, inhibiting growth by up to 80.3% (Figure 1). The strain also had a wide antibacterial spectrum and good inhibitory effect on all tested 11 fungal and 4 bacterial pathogens, with fungal colony growth inhibition rates of 50.1–71.1% (Table 1 and Figure 2), and the inhibition zone against pathogenic bacteria ranging 8.6–24.2 mm (Table 2 and Figure 3).

**Table 1:** Antifungal spectrum of strain T226 against plant pathogenic fungi.

Pathogenic fungi	Inhibition rate (%)	Statistical significance
*Rhizoctonia solani*	71.1 ± 1.8	a
*Colletotrichum gloeosporioides* “B”	68.3 ± 1.7	bc
*Colletotrichum gloeosporioides* “A”	69.0 ± 1.9	b
*Cochliobolus heterostrophus*	67.5 ± 2.1	c
*Colletotrichum musae*	65.1 ± 1.6	d
*Exserohilum turcicum*	63.4 ± 2.0	e
*Alternaria alternata*	62.7 ± 1.5	e
*Alternaria musae*	60.4 ± 2.2	f
*Curvularia lunata*	53.8 ± 1.7	g
*Sclerotium rolfsii*	50.1 ± 2.3	h

Data represent averages of three replicates. Lowercase letters within a column indicate significant differences (*p* < 0.05). *Colletotrichum gloeosporioides* “B” causing chili anthracnose, *Colletotrichum gloeosporioides* “A” causing star fruit tree anthracnose.

**Table 2:** Antifungal spectrum of strain T226 against plant pathogenic bacteria.

Pathogenic bacteria	Inhibition zone diameter (mm)	Statistical significance
*Xanthomonas campestris* pv. *citri*	24.4 ± 0.7	a
*Xanthomonas oryzae* pv. *oryzae*	15.3 ± 0.5	b
*Ralstonia solanacearum*	10.3 ± 0.6	c
*Pectobacterium carotovorum* pv. *carotovorum*	8.6 ± 0.4	d

Data represent averages of three replicates. Lowercase letters within a column indicate significant differences (*p* < 0.05).

#### 3.1.2 Strain T226 fermentation broth antibacterial activity

**Figure 4:**
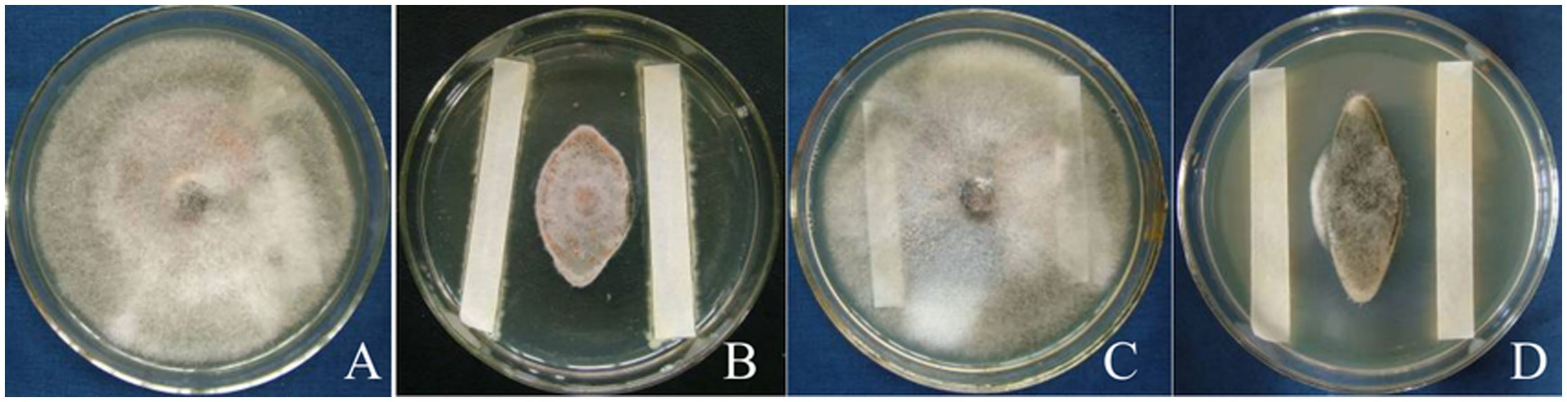
Effect of treatments on suppressive activity of strain T226 in fermentation fluids against citrus anthracnose pathogen. **(A)** Sterilized water (control). **(B)** Original fermentation fluid. **(C)** High-temperature-sterilized treatment. **(D)** Bacterium-free treatment.

Using *C. gloeosporioides* as an indicator bacterium, the biological activities in three fermentation broths (A–D) were determined. The fermentation broth of strain T226 showed no antagonistic activity after high temperature treatment at 121 °C for 20 min (Figure 4C); the antagonistic activity of the sterile supernatant (Figure 4D) was stronger than that of the fermentation broth (Figure 4B). High temperature inactivates antibacterial active substances in T226 strain fermentation broth.

#### 3.1.3 Effect of strain T226 on mycelial growth of citrus anthracnose pathogen

**Figure 5:**
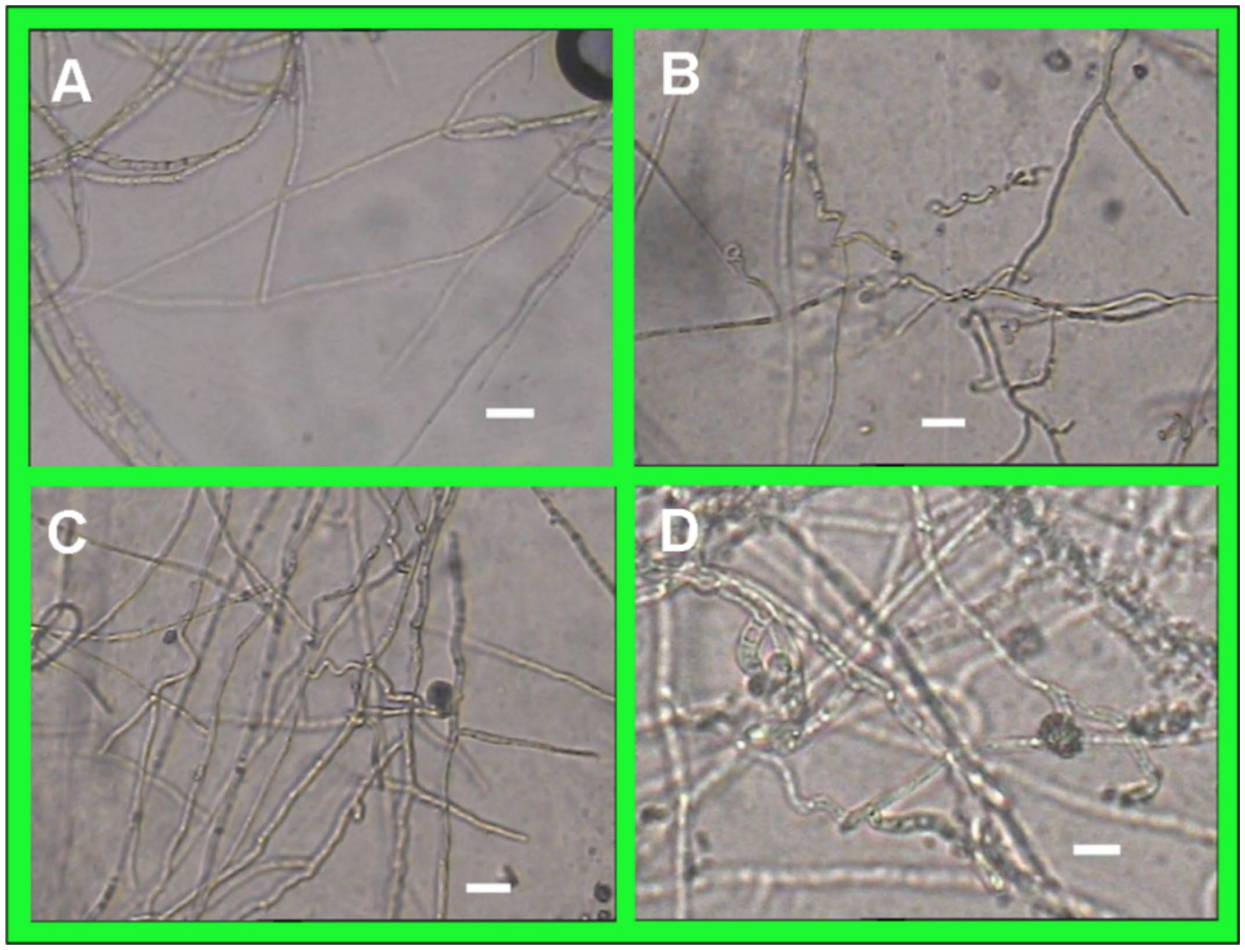
Morphological comparison between normal and abnormal hyphae of *C. gloeosporioides* (scale bars = 10 μm). **(A)** Hpyhal shape of *C*. *gloeosporioides* growing for 7 days. **(B–D)** Hpyhal shape of *C*. *gloeosporioides* coincubated on the media with strain T226 for 7 days.

After 7 days of confrontation culture with the antagonistic bacteria, the mycelial growth of *C. gloeosporioides* was significantly inhibited, and the mycelial growth at the edge of the pathogen colony was sparse. Compared with the control, strain T226 could cause the mycelium of *C. gloeosporioides* to be twisted, constricted or enlarged, and produce blisters. The protoplasts were condensed, and some of the mycelium were dissolved, so that the condensed protoplasts overflowed, and the mycelium was broken into segments (Figures 5A–C).

### 3.2 Stability of antibacterial activity of strain T226

**Figure 6:**
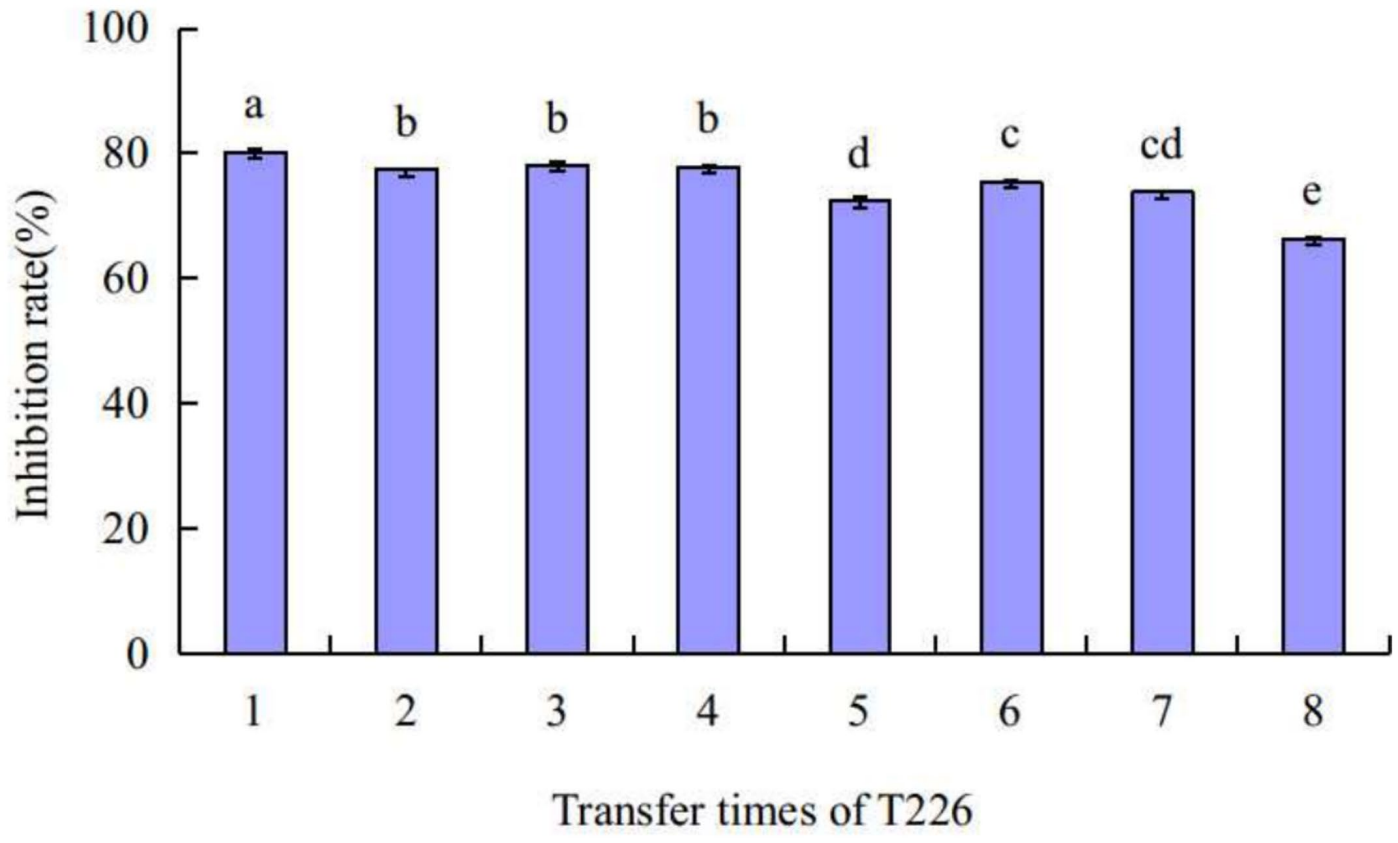
Stability of strain T226 in antimicrobial activity. Inhibition rates measured by dual-culture assay. Lowercase letters indicate significant differences between passages (*p* < 0.05).

The average antibacterial rate of strain T226 was 75.2% after eight continuous transplantations; the antibacterial rate for each transplantation time differed. After transplantation 5, the bacteriostatic rate decreased by ~5% points before tending to stabilize (Figure 6). There was no loss of bacteriostatic activity of strain T226 in transplantation culture.

### 3.3 Efficacy of strain T226 against postharvest citrus anthracnose

#### 3.3.1 Efficacy of wound inoculation

**Figure 7:**
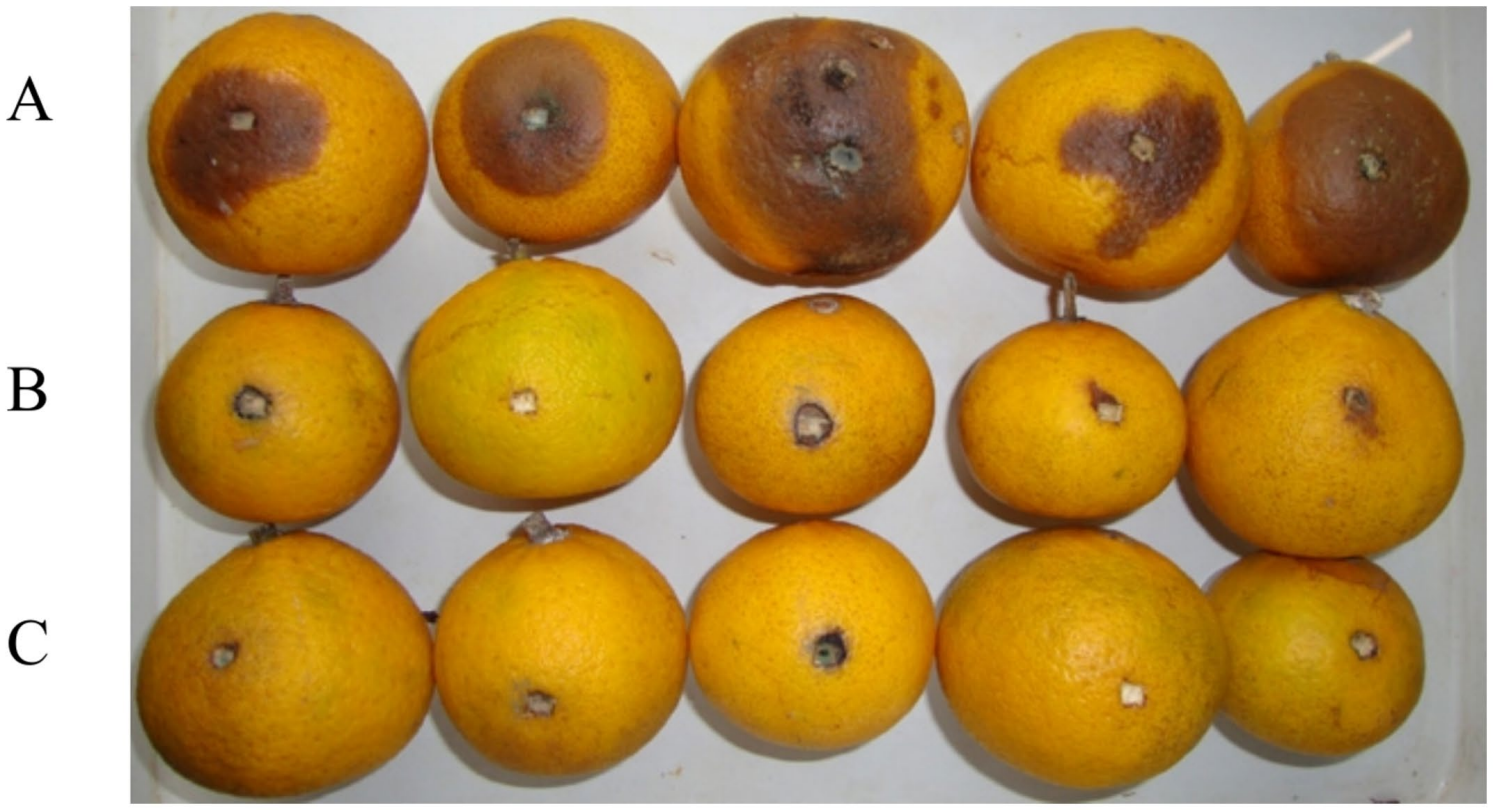
Inhibitory effects of citrus postharvest anthracnose by strain T226. **(A)** Control. **(B)** Strain T226. **(C)** Prochloraz.

After inoculation and 20 days storage at room temperature, the incidence of citrus anthracnose in control fruit reached 93.3%, while in the biocontrol strain T226 suspension (10^8^ cfu/mL) this was 20.0% (which differed significantly from the control (*p* < 0.05)). After 20 days of storage, the disease index of the control area reached 44.1, and most fruit had rotted. The disease index of the biocontrol strain T226 treatment area differed significantly, at 9.6. Control efficacy of strain T226 (78.2%) differed significantly from prochloraz (85.7%) at *p* < 0.05 (Table 3 and Figure 7).

**Table 3:** Efficacies of treatments to control artificially inoculated citrus anthracnose.

Treatment	Fruit rot incidence (%)	Disease index	Control efficacy (%)
T226	20.0 ± 1.8b	9.6 ± 1.1b	78.2 ± 2.5b
Prochloraz	16.7 ± 1.5b	6.3 ± 0.8b	85.7 ± 2.1a
CK	93.3 ± 2.1a	44.1 ± 3.2a	—

#### 3.3.2 Control effect of natural infection

**Figure 8:**
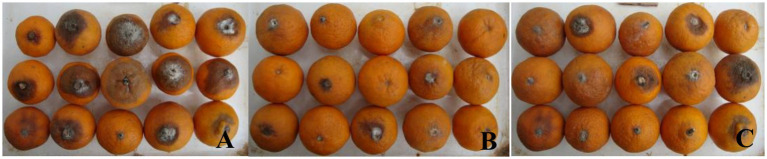
Efficacies of antagonist applications against postharvest citrus naturally infected by anthracnose. **(A)** Control (CK). **(B)** Strain T226. **(C)** Prochloraz.

After soaking in treatment solution and storage at room temperature for 60 days, rates of rot in fruit treated with antagonistic strain T226 and prochloraz differed significantly, at 26.7 and 36.7%, respectively; both treatments performed significantly better than the control (73.3%). The control effect of strain T226 on postharvest citrus anthracnose (61.3%) exceeded that of prochloraz (49.1%), and differences between them were significant (Table 4 and Figure 8).

**Table 4:** Efficacies of treatment to control naturally infections of citrus by anthracnose.

Treatment	Fruit rot incidence (%)	Disease index	Control efficacy (%)
T226	26.7 ± 3.1c	24.8 ± 2.8b	61.3 ± 4.2a
prochloraz	36.7 ± 3.5b	32.6 ± 3.1b	49.1 ± 4.8b
CK	73.3 ± 4.2a	64.1 ± 4.5a	/

#### 3.3.3 Identification of strain T226

**Figure 9:**
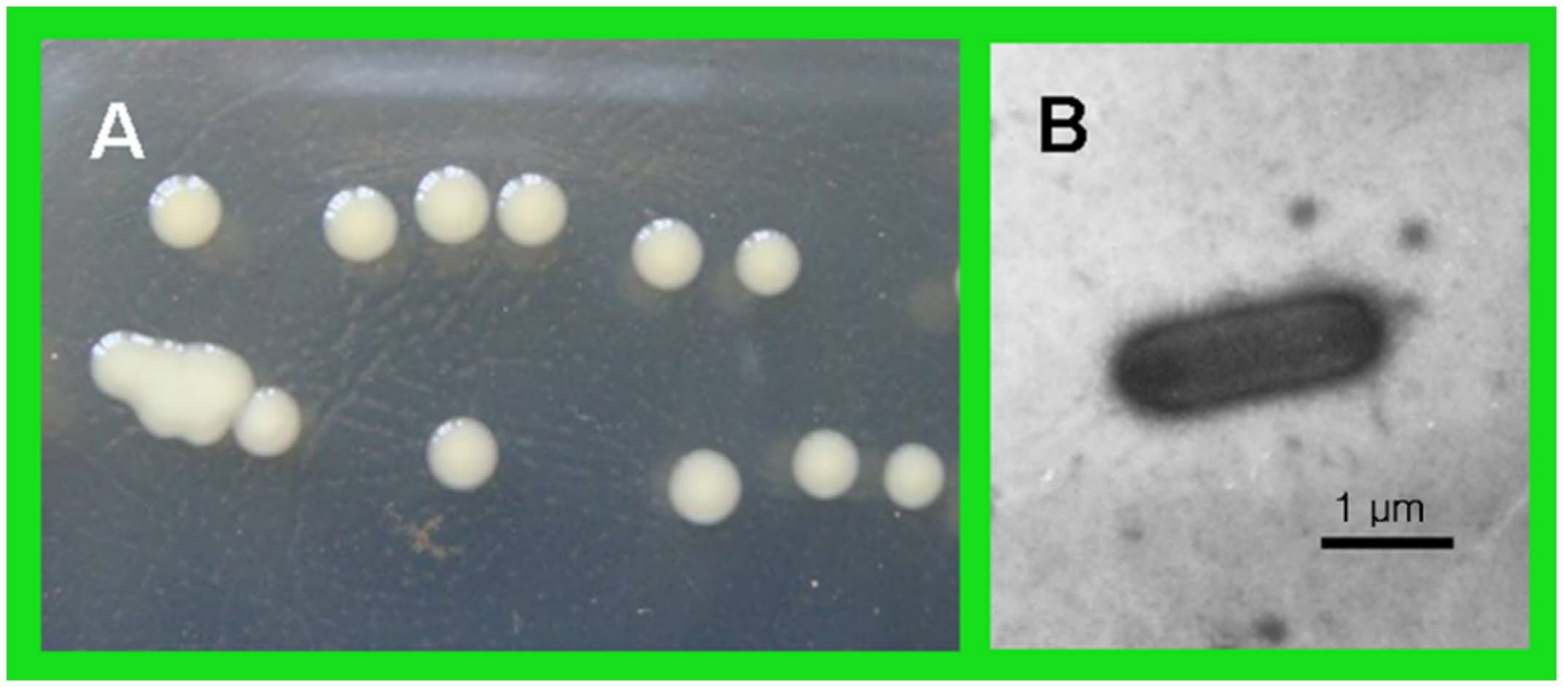
Morphology of strain T226. **(A)** Individual colonies. **(B)** A bacterial cell.

The antagonistic bacterium T226 was rod-shaped, 1.9–2.1 μm long, 0.6–0.8 μm wide, single, lacked a flagellum, produced mucus, had a sliding movement, and was Gram-negative. The growth temperature was 10–37 °C (optimum 31 °C) and the growth pH was 3–12 (optimum 5.0). After 2 days of cultivation at 28 °C on NA plates, colonies were round, of diameter ~2 mm, had smooth surfaces with thin edges, and formed by sliding motion. Colonies were moist, sticky, light yellow in color, and produced melanin. After 5 days, colonies were yellowish brown (Figure 9).

Available carbon sources for strain T226 included glucose, mannitol, L-arabinose, trehalose, and lactose; unusable carbon sources included maltose, soluble starch, D-galactose, inulin, and xylitol (Table 5). Available nitrogen sources included L-lysine, L-leucine, L-histidine, L-cysteine, ammonium nitrate, urea, ammonium sulfate, and ammonium chloride; DL-aspartic acid was an unavailable nitrogen source (Table 5).

**Table 5:** Carbon source uses by antagonistic strains.

Carbon	Utilization	Nitrogen	Utilization
Glucose Maltose Soluble amylum D-galactose Inulin Mannose L-arabinose Xylitol Trehalose Lactose	+ − − − − + + − + +	L-Lysine L-cystine DL-Aspartic acid L-Histidine L-cysteine Ammonium nitrate Urea Ammonium sulphate Ammonium chloride	+ + − + + + + + +

“+” is positive and “−” negative.

Positive physiological and biochemical reactions included starch hydrolysis, hydrogen sulfide test, Tween 80, citrate utilization, glucose oxidation to produce acid, gelatin hydrolysis, contact enzyme, and aerobic; negative physiological and biochemical reactions included the M.R. test, indole test, and oxidase. Biocontrol bacteria could grow in 2% sodium chloride solution, but not in >3% sodium chloride solution (Table 6).

**Table 6:** Physiological and biochemical reactions of antagonistic strains.

Item	Reaction	Item	Reaction
Starch hydrolysis M.R. H_2_S test Indole product Tween 80 Sodium hydrolysis D-glucose Gelatin liquefaction	+ − + − + + − +	Oxidase Contact enzyme Aerobic Growth in 2% NaCl Growth in 3.5% NaCl Growth in 5% NaCl Growth in 7% NaCl Growth in 10% NaCl	− + + + − − − −

“+” is positive and “−” negative.

**Figure 10:**
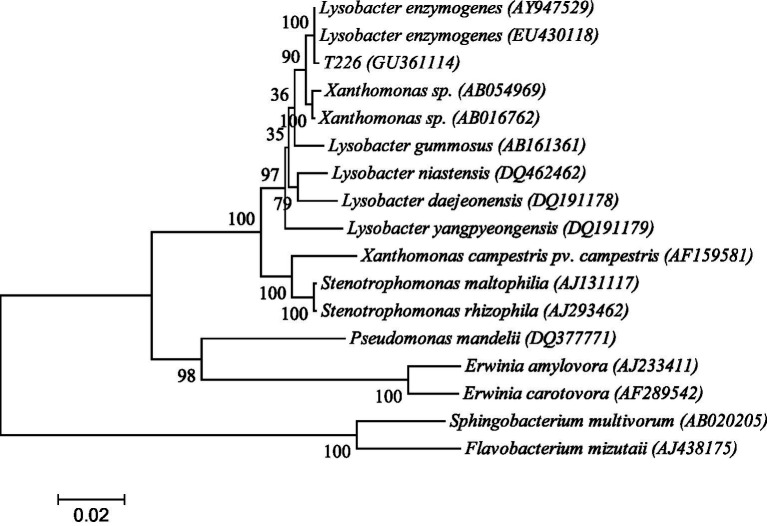
Phylogenetic tree based on 16S rDNA sequence homology of strain T226 with related bacteria on GenBank. Numbers in parentheses (GenBank sequence accession numbers). Numbers at branch points (percentages supported by bootstrap). The scale bar represents 0.02 substitutions per nucleotide position.

The 16S rDNA genome of strain T226 was amplified by using primers PF1 and PR1; a 16S rDNA fragment of ~1.5 kb was obtained. Sequencing results revealed a high homology with *Lysobacter*, with a maximum homology between strain T226 and *L. enzymogenes* (accession number: EU430118) of 99%. The strain T226 sequence is registered in GenBank (accession number GU361114). The phylogenetic tree based on 16S rDNA sequences of strain T226 and related bacteria from GenBank was constructed using MEGA version 4.0 (Tamura et al., 2021) (Figure 10). Strain T226 clusters with two *L. enzymogenes* at the 100% bootstrap support.

We identify strain T226 as *L. enzymogenes* based on a combination of morphology (Figure 8), physiology and biochemistry (Tables 5, 6), and genetic (16S rDNA) results (Figure 9).

## 4 Discussion

This study demonstrates that *Lysobacter enzymogenes* T226, isolated from sugarcane rhizosphere soil, is a highly effective and stable biocontrol agent against postharvest citrus anthracnose caused by *Colletotrichum gloeosporioides*. The following discussion will focus on its taxonomic position, broad-spectrum activity, functional mechanisms, and potential for commercial development, contextualizing our findings within the existing literature on biocontrol.

### 4.1 Taxonomic identification and novelty of *Lysobacter enzymogenes* T226

The polyphasic identification confirmed strain T226 as *L. enzymogenes*, characterized by its rod-shaped morphology, sliding motility, and production of melanin in aging cultures (Figure 9), which are classic traits of this species (Christensen and Cook, 1978). The 16S rDNA phylogenetic analysis provided definitive evidence, with 99% homology to the type strain (Figure 10). Most significantly, to our knowledge, this is the first report of an *L. enzymogenes* strain exhibiting potent activity against citrus postharvest anthracnose. While *Lysobacter* spp. are renowned for controlling soil-borne diseases (Wang et al., 2023), its application against postharvest fruit pathogens remains largely unexplored, highlighting the novelty of our isolate.

### 4.2 Broad-spectrum antimicrobial activity and its implications

Beyond its efficacy against *C. gloeosporioides*, strain T226 displayed a surprisingly broad inhibition spectrum against 10 fungi and 4 bacteria (Tables 1, 2 and Figures 2, 3). This broad-spectrum activity is a highly desirable trait for a commercial biocontrol agent, as it suggests T226 could be deployed against a complex of postharvest pathogens (e.g., *Penicillium* spp., *Alternaria* spp.) commonly co-occurring on citrus fruit (Solanki et al., 2024). This advantage positions T226 favorably against niche-specific biocontrol agents like *Candida oleophila*, which primarily targets *Penicillium* spp. (Droby et al., 2002). The ability to also inhibit bacterial pathogens such as *Xanthomonas citri* further expands its potential utility.

### 4.3 Antagonistic stability and *in vivo* biocontrol efficacy

A critical requirement for the commercialization of a microbial agent is its genetic and functional stability. Our data clearly show that T226 maintained a high level of antagonistic activity (~75%) even after eight successive subcultures (Figure 6). This metabolic stability suggests a low risk of rapid trait degeneration during large-scale fermentation, a common hurdle for other biocontrol bacteria. This stability translated into consistent *in vivo* efficacy. In artificial inoculation assays, T226 achieved a control efficacy of 78.2%, approaching the performance of the chemical fungicide prochloraz (85.7%) (Table 3). More importantly, under natural infection conditions, which better simulate commercial storage scenarios, T226’s efficacy (61.3%) surpassed that of prochloraz (49.1%) after 60 days (Table 4). This superior long-term performance under natural conditions underscores its strong potential for practical application.

### 4.4 Proposed mechanisms of antagonistic action

Our results provide insights into the possible mechanisms behind T226’s antagonism. The loss of activity in heat-treated fermentation broth (Figure 4C) strongly indicates that the primary antifungal metabolites are heat-labile, likely proteinaceous antibiotics or complex macrocyclic compounds, such as the Heat-Stable Antifungal Factor (HSAF) commonly produced by *L. enzymogenes* (Qian et al., 2009; Chen et al., 2018). This is corroborated by the clear morphological damage observed in the fungal hyphae, including swelling, vacuolation, and lysis (Figure 5). These symptoms are typical of the action of lytic enzymes and secondary metabolites that target the cell membrane and wall. The retained activity in the cell-free supernatant (Figure 4D) confirms that the inhibitory compounds are diffusible.

### 4.5 Limitations and future research directions

Despite the promising results, several limitations must be addressed to translate T226 into a commercial product. First, we should focus on optimizing fermentation conditions, including determining the optimal carbon and nitrogen sources for both growth and antimicrobial compound yield, to facilitate its potential development into a bio-fungicide. Second, the exact identity and biosynthesis pathways of the active metabolites remain unknown. Future work should involve genomic sequencing to identify the gene clusters responsible (e.g., for HSAF production) and purification of the compounds. Third, as with many non-spore-forming bacteria, the formulation of T226 to enhance its shelf-life and tolerance to environmental stresses (e.g., UV, desiccation) is a major challenge (Segarra et al., 2015). Research into microencapsulation or clay-based carriers is warranted. Finally, a comprehensive assessment of its ecological impact on the native fruit microbiome and its safety for consumers is essential before field deployment.

## 5 Conclusion

This study establishes *Lysobacter enzymogenes* T226 as a novel and promising biocontrol agent against citrus postharvest anthracnose. Its identification as a potent antagonist of *Colletotrichum gloeosporioides*, coupled with its broad-spectrum activity and high *in vitro* stability, underscores its significant potential for development into a sustainable alternative to synthetic fungicides. These findings provide a strong foundation for the future application of *L. enzymogenes* in integrated postharvest management strategies.

## Data Availability

The datasets presented in this study can be found in online repositories. The names of the repository/repositories and accession number(s) can be found in the article/supplementary material.

## Footnotes

^1^ www.ncbi.nlm.nih.gov
